# Impact of sewage water irrigation on *Datura innoxia* grown in sandy loam soil

**DOI:** 10.1186/s12870-022-03935-9

**Published:** 2022-12-02

**Authors:** Amany H. A. Abeed, Mohammed Ali, Mamdouh A. Eissa, Suzan A. Tammam

**Affiliations:** 1 Department of Botany and Microbiology, Faculty of Science, Assiut University, Assiut, 71516 Egypt; 2 Egyptian Deserts Gene Bank, North Sinai Research Station, Department of Genetic Resources, Desert Research Center, Cairo 11753, Egypt, Desert Research Center, Cairo, 11753 Egypt; 3 Department of Soils and Water, Faculty of Agriculture, Assiut University, Assiut, 71526 Egypt

**Keywords:** Alkaloids, *Datura innoxia*, nitrate reductase, Phenylalanine ammonia lyase, Proline, Sewage water

## Abstract

**Background:**

A potential solution for recycling and reusing the massively produced sewage water (SW) is to irrigate certain plants instead of highly cost recycling treatment. Although the extensive and irrational application of SW may cause environmental pollution thus, continual monitoring of the redox status of the receiver plant and the feedback on its growth under application becomes an emergent instance. The impact of SW, along with well water (WW) irrigation of medicinal plant, *Datura innoxia*, was monitored by some physio-biochemical indices.

**Results:**

The SW application amplified the growth, yield, minerals uptake, and quality of *D. innoxia* plants compared to the WW irrigated plants. The total chlorophyll, carotenoid, non-enzymatic antioxidants, viz. anthocyanin, flavonoids, phenolic compounds, and total alkaloids increased by 85, 38, 81, 50, 19, and 37%, respectively, above WW irrigated plants. The experiment terminated in enhanced leaf content of N, P, and K by 43, 118, and 48%, respectively. Moreover, stimulation of carbon and nitrogen metabolites in terms of proteins, soluble sugars, nitrate reductase (NR) activity, and nitric oxide (NO) content showed significant earliness in flowering time. The SW application improved not only *Datura* plants’ quality but also soil quality. After four weeks of irrigation, the WW irrigated plants encountered nutrient deficiency-induced stress evidenced by the high level of proline, H_2_O_2_, and MDA as well as high enzyme capabilities. Application of SW for irrigation of *D. innoxia* plant showed the improvement of secondary metabolites regulating enzyme phenylalanine ammonia-lyase (PAL), restored proline content, and cell redox status reflecting high optimal condition for efficient cellular metabolism and performance along the experiment duration.

**Conclusions:**

These evidences approved the benefits of practicing SW to improve the yield and quality of *D. innoxia* and the feasibility of generalization on multipurpose plants grown in poor soil.

## Background

In 1966, resources of renewable water were 2189 m^3^/capita/year, which will minimize to 500 m^3^/capita/year by 2025 [1], obligatory, it requires us to rationalize consumption. The agriculture prerequisites surpass 80 % of the total water demand [2]. The development of Egypt’s economy intensely relies on its capacity to manage and preserve its water resources to face the predicted elevation in water demand from other sectors, including industrial and municipal water supply. SW represents a continuous disposable source of water [3, 4]. Vast effluent quantities are produced as a consequence of large-scale industrialization and urbanization [5]. At present, Egypt generates an estimated 5.5–6.5 billion cubic meter (BCM) of SW per year, accounted as 2.5% of water resources in Egypt [5]. Of that amount, only 0.7 BCM annually is utilized in agriculture, chiefly in direct as well as indirect reuse in desert areas via mixing with the water of agricultural drainage [6]. Since 1980, this practice has increased as tremendous potential relevance to Egypt. SW is primarily rich in organic matter (OM), macro- and micronutrients, dissolved minerals, and irrigation with SW consequently increases soil fertility and nutrient content [7, 8]. However, the over-application of SW may cause chemical pollution problems, especially for edible crop plants, due to accumulation of heavy metals (HMs) [8] and increase sodicity as well as soil salinity [9]. Moreover, when SW is continually utilized as a sole source of irrigation water, toxic chemicals and excessive nutrients could be applied to plants, which would impose undesirable and toxic effects on plant productivity and quality. Despite all the aforementioned issues, SW reuse is necessary to design future water policies. This makes it mandatory to critically evaluate the effects of this practice on agriculture, human health, and the environment [7].

The Egyptian government is rising significant concerns about human health from SW-irrigated crops; thus, inedible plants (grasses and forest trees) have been replaced compared to crops grown in control, where SW was not previously applied [5]. Using SW in the agriculture sector might solve the expected energy crisis [10]. Oil-accumulating plants (sunflower, corn, olive, and castor) and carbohydrate-accumulating plants (wheat, rice, corn, sugarcane, and potatoes) that might be cultivated for hydrogen and methane production, or bioethanol or biodiesel production, trees grown for greenery at hotels and touristic villages, nursery plant, fiber crops, industrial oil crops, wood trees, fodder/ feed crops, trees for city’s green belts and roads or high ways afforestation, all seem to be most profitable alternatives [5]. The private sector and land reclamation are cultivating the aforementioned plants in several country sites, particularly in the south, which are deliberately constructed to convert low-quality land (e.g., sand) and low-quality water (e.g., sewage) into valuable resources [10]. In Egypt, many areas currently lack the high capability for agriculture due to poor nutritional availability and high calcium carbonate content [11]. Therefore, it is necessary to explore different available practices, assess their potentiality for viable agriculture, and plan appropriate sustainable land use. Accordingly, SW application has a double privilege, where it is a costless alternative irrigation water source and achieves environmentally SW disposal or recycling management as well as improving the quality of the low-grade soil with poor input of OM by increasing soil nitrogen, phosphorus, and potassium.

Recently, the growth of medicinal plants such as *Datura* received high awareness nationally [12]. Many species of *Datura* were cultivated for secondary metabolites production. *Datura innoxia*, a perennial plant with inedible parts, is a commercially important plant for having a broad range of heavy market demand for bioactive metabolites due to their extensive use in medicine, where its leaves contain tropane alkaloids with pharmacological activities and significant medicinal properties such as atropine, scopolamine, and hyoscyamine that are used as parasympathicolitics as having the ability to suppress the parasympathetic nerve activity [13, 14]. Plants grow more luxuriously when levels of N fertilizer increase in the medium and alkaloid synthesis extent paralleled plant overall growth [15]. Some previous studies indicated that SW irrigation can enhance plant growth, yield, and quality while increasing nutrient availability [4, 9, 16]. This practice became popular in countries that faced water scarcity especially Egypt. However, the extensive and irrational use of SW for irrigation practices imposes significant disadvantages including plant growth and performance disorders due to the highly loaded HMs per unit of time and/or the ionic stress. Hence, the impact of sewage water irrigation should be monitored keeping in view for how long SW application can be effective and safely operational. Some physio-biochemical indices can be monitored being a mirror of the plant performance efficacy under SW irrigation. The current study aimed to address the impact of SW practiced on *Datura* via following up some growth parameters in terms of plant dry weight (DW), branches, and a number of flowers, besides pigments content, secondary metabolites content, nutritional profile, and redox status in terms of stress markers and membrane damage traits, as well as malondialdehyde (MDA) as a lipid peroxidation marker, H_2_O_2,_ and its quenching enzymes. Moreover, the study of antioxidant response in addition to metabolites in the inedible commercially valuable plant, *Datura innoxia*, may collectively help donating new reference information for underlining the possibility of practicing SW and the feasibility of generalization on large-scale plants. Thus, plant growth assessments and the characterization of enzymatic as well as non-enzymatic antioxidant responses were parallel conducted. This research also investigated the changes in soil physicochemical parameters as a secondary objective.

### Experimental results

#### Physical and Chemical Characteristics of Sewage Water

Data shown in Table 1 revealed that pH and EC concentration in SW were higher (7.4 and 0.80 ds m^− 1^, respectively) than that of WW (7.2 and 0.51 ds m^− 1^, respectively). Moreover, almost all measured nutrients determined in the SW samples collected from the Arab Elmadabegh sewage line were higher than the corresponding criteria of the WW (tap water). However, the micronutrients and HMs in the SW herein are relatively low and within the limits of standard concentrations.

**Table 1:** Properties of well water and sewage water used in irrigation

Properties	Well water	Sewage water
pH	7.20 ± 0.43	7.41 ± 0.22
EC (dS/m)	0.51 ± 0.04	0.80 ± 0.05
Na (mg/L)	0.66 ± 0.05	1.22 ± 0.07
K (mg/L)	0.21 ± 0.01	0.54 ± 0.03
Mg (mg/L)	0.69 ± 0.01	1.81 ± 0.08
Ca (mg/L)	2.76 ± 0.07	5.1 ± 0.10
CO_3_ (mg/L)	0.4 ± 0.01	0.9 ± 0.02
HCO_3_ (mg/L)	2.2 ± 0.03	4.9 ± 0.11
Cl (mg/L)	1.61 ± 0.02	4.2 ± 0.06
Fe (mg/L)	18 ± 0.92	83 ± 0.83
Zn (mg/L)	2 ± 0.09	2 ± 0.10
Cu (mg/L)	1.2 ± 0.07	2.4 ± 0.11
Cd (mg/L)	nd	0.07 ± 0.002
Pb (mg/L)	0.9 ± 0.01	1.5 ± 0.07
Mn (mg/L)	16 ± 0.66	22 ± 0.77

*V* Volume, *EC* Electrical conductivity, *dS/m* decisiemens per metre, *mg* Milligram, *g* Gram, *kg* Kilogram, *L* Liter, *nd* Not detected. Each value is an average of 4 replicates

#### Physical and Chemical Characteristics of the Soil

Physico-chemical characteristics of the soil were interpreted in Table 2. The results showed that the used soil was slightly alkaline (pH 8.02) with sandy loam textural grade having little concentration of OM (11 g/kg) and poor in nitrogen (260 mg/kg) and phosphorus content (2.5 mg/kg).

**Table 2:** Physico-chemical characteristics of soil used in the study

Soil properties	Value
Sand (g/kg)	76 ± 0.57
Clay (g/kg)	14 ± 0.31
Silt (g/kg)	10 ± 0.22
Texture class	Sandy loam
pH	8.02 ± 0.08
EC (dS/m)	1.85 ± 0.02
Organic carbon (g/kg)	15 ± 0.12
Total nitrogen (mg/kg)	500 ± 0.86
Organic matter (g/kg)	10.9 ± 0.21
Total phosphorus (P) (mg/kg)	293 ± 3.21
Na (mg/L)	1.69 ± 0.05
K (mg/L)	0.56 ± 0.01
Mg (mg/L)	0.62 ± 0.02
Ca (mg/L)	3.11 ± 0.11
CO_3_ (mg/L)	5.59 ± 0.15
HCO_3_ (mg/L)	7.34 ± 0.21
Cl (mg/L)	3.03 ± 0.22
Available Fe (mg/kg)	3.85 ± 0.20
Available Zn (mg/kg)	1.96 ± 0.08
Available Cu (mg/kg)	0.75 ± 0.013
Available Mn (mg/kg)	1.93 ± 0.06
Available Cd (mg/kg)	Nd
Available Pb (mg/kg)	Nd

*V* Volume, *EC* Electrical conductivity, *dS/m* decisiemens per metre, *g* Gram, *kg* Kilogram, *nd* Not detected

Data depicted in Table 3 revealed that the soil pH reduced with SW irrigation, reaching a value of 6.9. Whereas, the soil concentrations of OM, organic carbon (OC), N, and P recorded higher values by SW irrigation of 5.9, 11.3, 0.33, and 0.28 g/Kg, respectively. Obviously, for Fe, Zn, Cu, and Mn, SW treatments increased soil micronutrients as well as macronutrients. However, the HMs (Cd and Pb) were not affected by SW irrigation.

**Table 3:** Chemical characteristics of soil used in the study at the end of experiment

Properties	Irrigated by well water	Irrigated by sewage water
pH	7.90 ± 0.12	6.90 ± 0.11
EC (dS/m)	0.31 ± 0.01	3.50 ± 0.10
Organic matter (g/kg)	9.80 ± 0.15	13 ± 0.34
Organic carbon (g/kg)	11 ± 0.21	16.33 ± 0.44
Total nitrogen (N) (mg/Kg)	240 ± 0.76	433 ± 0.88
Total phosphorus (P) (mg/kg)	220 ± 0.77	311 ± 0.98
Na (mg/L)	0.56 ± 0.02	11.22 ± 0.42
K (mg/L)	0.21 ± 0.007	5.14 ± 0.12
Mg (mg/L)	0.52 ± 0.01	8.41 ± 0.14
Ca (mg/L)	2.06 ± 0.11	14.10 ± 0.43
CO_3_ (mg/L)	0.35 ± 0.01	2.90 ± 0.11
HCO_3_ (mg/L)	1.60 ± 0.10	7.90 ± 0.15
Cl (mg/L)	1.61 ± 0.10	11.21 ± 0.46
Fe (mg/L)	18 ± 0.66	70 ± 0.55
Zn (mg/L)	2 ± 0.11	12 ± 0.40
Cu (mg/L)	1.20 ± 0.09	1.81 ± 0.09
Cd (mg/L)	nd	Nd
Pb (mg/L)	0.09 ± 0.002	0.13 ± 0.02
Mn (mg/L)	9 ± 0.22	18 ± 0. 43

*V* Volume, *EC* Electrical conductivity, *dS/m* decisiemens per metre, *mg* Milligram, *g* Gram, *kg* Kilogram, *L* Liter, *nd* Not detected. Each value is an average of 4 replicates

#### Growth parameters and physio-biochemical alternations of *Datura innoxia* Plant *Datura innoxia* Dry Weight, Photosynthetic Pigments, No. of Flowers and Lateral Branches, Primary Metabolites and Proline Content Under Sewage Water Irrigation

The obtained results in Table 4 revealed that, by the end of the experiment duration, the SW application significantly (*p* < 0.0001) increased plant biomass in terms of DW with a percent increase amounted to 163%, while plants irrigated with WW increased by only 26% when compared to corresponding starter values (1.33 and 1.35, respectively).

**Table 4:** Measurements of different growth parameters of *Datura innoxia* plant under two treatments: well water (WW) or sewage water (SW) for 10 weeks. Each value represents a mean value of four replicates ±SE. The observations were recorded on 2 randomly selected plants on plot mean basis analysis per replication per treatment for each correspond week

Water source	Weeks	DW	BN	FN	TC	Car	An	Fl	Ph	Al	Pro
**SW**	**SV**	1.33 ± 0.05	–	–	1.22 ± 0.05	1.83 ± 0.02	0.43 ± 0.03	51.00 ± 1.47	9.50 ± 0.21	0.90 ± 0.01	1.71 ± 0.05
	**1st**	1.57 ± 0.01	–	–	1.72 ± 0.02	1.89 ± 0.04	0.50 ± 0.02	58.00 ± 0.71	10.00 ± 0.71	1.31 ± 0.04	3.25 ± 0.25
	**2nd**	1.86 ± 0.02	–	–	1.98 ± 0.04	1.97 ± 0.02	0.56 ± 0.01	66.00 ± 1.47	14.00 ± 0.41	1.77 ± 0.03	3.50 ± 0.29
	**3rd**	1.89 ± 0.04	–	–	2.20 ± 0.11	1.99 ± 0.03	0.70 ± 0.01	71.00 ± 0.91	18.00 ± 0.41	1.98 ± 0.05	4.25 ± 0.25
	**4th**	2.11 ± 0.08	1.00 ± 0.25	2.00 ± 0.25	2.50 ± 0.18	2.60 ± 0.13	0.78 ± 0.00	77.00 ± 0.91	23.00 ± 0.41	2.50 ± 0.20	2.75 ± 0.25
	**5th**	2.65 ± 0.05	2.00 ± 0.25	3.00 ± 0.25	2.89 ± 0.11	2.65 ± 0.05	0.82 ± 0.02	80.00 ± 0.91	29.00 ± 0.91	2.87 ± 0.07	3.00 ± 0.41
	**6th**	2.77 ± 0.10	2.00 ± 0.48	3.00 ± 0.25	3.03 ± 0.02	2.78 ± 0.10	0.89 ± 0.02	87.00 ± 0.58	34.00 ± 0.41	3.33 ± 0.14	1.70 ± 0.10
	**7th**	2.89 ± 0.03	3.00 ± 0.25	4.00 ± 0.25	3.40 ± 0.14	2.89 ± 0.35	0.94 ± 0.02	92.00 ± 1.29	39.00 ± 0.71	3.72 ± 0.08	2.00 ± 0.41
	**8th**	3.05 ± 0.05	3.00 ± 0.25	4.00 ± 0.25	3.63 ± 0.09	3.44 ± 0.11	0.97 ± 0.06	96.00 ± 1.08	44.00 ± 0.41	4.00 ± 0.15	1.63 ± 0.14
	**9th**	3.30 ± 0.15	4.00 ± 0.25	5.00 ± 0.41	3.71 ± 0.10	3.63 ± 0.09	1.11 ± 0.06	112.00 ± 1.22	53.00 ± 1.08	4.51 ± 0.16	1.50 ± 0.02
	**10th**	3.50 ± 0.18	4.00 ± 0.25	7.00 ± 0.41	3.72 ± 0.13	4.55 ± 0.21	1.50 ± 0.02	123.00 ± 1.22	57.00 ± 1.47	5.93 ± 0.17	1.70 ± 0.04
**WW**	**SV**	1.35 ± 0.05	–	–	1.24 ± 0.04	1.81 ± 0.04	0.44 ± 0.02	51.50 ± 1.76	9.00 ± 0.41	0.90 ± 0.01	1.54 ± 0.04
	**1st**	1.45 ± 0.06	–	–	1.51 ± 0.02	1.84 ± 0.05	0.47 ± 0.01	52.50 ± 1.76	10.00 ± 0.41	0.96 ± 0.02	1.54 ± 0.04
	**2nd**	1.61 ± 0.06	–	–	1.70 ± 0.04	1.93 ± 0.02	0.49 ± 0.01	52.25 ± 1.03	10.00 ± 0.71	1.11 ± 0.03	1.83 ± 0.05
	**3rd**	1.64 ± 0.03	–	–	1.75 ± 0.02	2.19 ± 0.12	0.51 ± 0.02	57.00 ± 0.82	11.00 ± 0.41	1.63 ± 0.14	2.04 ± 0.05
	**4th**	1.69 ± 0.02	0.00 ± 0.00	0.00 ± 0.00	1.79 ± 0.02	2.24 ± 0.20	0.56 ± 0.01	62.50 ± 1.76	19.25 ± 0.48	1.88 ± 0.03	3.25 ± 0.48
	**5th**	1.72 ± 0.03	1.00 ± 0.25	0.00 ± 0.00	1.83 ± 0.02	2.73 ± 0.11	0.59 ± 0.03	68.50 ± 0.65	20.00 ± 0.82	1.99 ± 0.07	4.25 ± 0.25
	**6th**	1.75 ± 0.02	2.00 ± 0.29	0.00 ± 0.00	1.92 ± 0.02	2.81 ± 0.16	0.60 ± 0.02	67.50 ± 0.65	26.25 ± 0.85	2.21 ± 0.23	6.00 ± 0.41
	**7th**	1.71 ± 0.02	2.00 ± 0.00	2.00 ± 0.25	1.90 ± 0.04	2.88 ± 0.3	0.64 ± 0.01	66.25 ± 1.55	32.50 ± 1.04	2.21 ± 0.24	6.00 ± 0.41
	**8th**	1.71 ± 0.02	2.00 ± 0.25	2.00 ± 0.29	1.83 ± 0.03	2.97 ± 0.18	0.77 ± 0.01	70.00 ± 0.41	36.25 ± 1.03	3.43 ± 0.10	7.00 ± 0.41
	**9th**	1.70 ± 0.03	2.00 ± 0.25	2.00 ± 0.41	1.80 ± 0.04	3.03 ± 0.33	0.79 ± 0.01	77.75 ± 1.60	41.50 ± 1.19	3.83 ± 0.05	6.25 ± 0.48
	**10th**	1.70 ± 0.04	2.00 ± 0.25	3.00 ± 0.25	1.83 ± 0.05	3.33 ± 0.15	0.83 ± 0.02	82.25 ± 1.60	48.25 ± 0.63	4.30 ± 0.18	8.00 ± 0.71
**L.S.D.(0.05)**		**0.13**	**0.41**	**0.43**	**0.15**	**0.33**	**0.06**	**2.45**	**1.51**	**0.25**	**0.63**
***P*** **Value**		**< 0.0001**	**< 0.0001**	**< 0.0001**	**< 0.0001**	**0.0032**	**< 0.0001**	**< 0.0001**	**< 0.0001**	**< 0.0001**	**< 0.0001**
**Source**	**df**	**Mean Square**									
		**DW**	**BN**	**FN**	**TC**	**Car**	**An**	**Fl**	**Ph**	**Al**	**Pro**
**Weeks**	10	1.393**	12.632**	21.257**	2.147**	3.741**	0.375**	2064.309**	1881.096**	13.933**	6.964**
**Water Sources**	1	14.378**	15.557**	70.920**	21.721**	1.078**	1.166**	7640.909**	804.045**	12.768**	78.001**
**Weeks x Water Sources**	10	0.805**	1.832**	5.170**	1.018**	0.342**	0.066**	298.109**	25.895**	0.487**	19.836**
**Error**	**66**	**0.017**	**0.170**	**0.186**	**0.023**	**0.111**	**0.002**	**6.000**	**2.296**	**0.060**	**0.394**

*SV* Starter value, *DW* Dry weight (g/plant), *BN* Branch number (branches/plant), *FN* Flowers/plant), *TC* Total chlorophyll (mg/g FW), *Car* Carotenoids (mg/g FW), *An* Anthocyanin (μmol/g FW), *FL* Flavonoids (mg/g FW), *Ph* Phenolics (mg/g FW), *Al* Alkaloids (mg/g FW), *Pro* Proline (mg/g FW)

Visibly, the application of SW irrigation had a potential impact on *Datura innoxia*’s morphological feature in terms of branching and flowering criterion. Early branching and flowering resulted within the 2nd and 4th week, respectively, submitted the highest number of branches (4 branches/plant) and flowers (7 flowers/plant) by the end of the experiment. In contrast, the WW irrigated plants exhibited branch initiation at 5th week and floral initiation at 7th week with final relatively low branch and flower number (2 branches and two flowers/plant).

SW application positively influenced the photosynthetic pigment synthesis in terms of the total chlorophyll and carotenoids over the corresponding values recorded in WW irrigated plants (Table 4) on the 10th week, with a percent increase of 102 and 38% for total chlorophyll and carotenoids, respectively.

Unlike the chlorophyll fluctuation, proline showed heterogeneous activities, and it was kept the baseline of control and restored by SW application along the study duration while it was promoted considerably by the end of the experiment with WW irrigation with a percent increase of 433% over the corresponding starter value (1.5 mg/g DW). The displayed data in Table 4 showed that the interaction between water application and time significantly impacted DW, branch and flower number, total chlorophyll, and proline content of *Datura* plants (*P* < 0.0001).

**Fig. 1:**
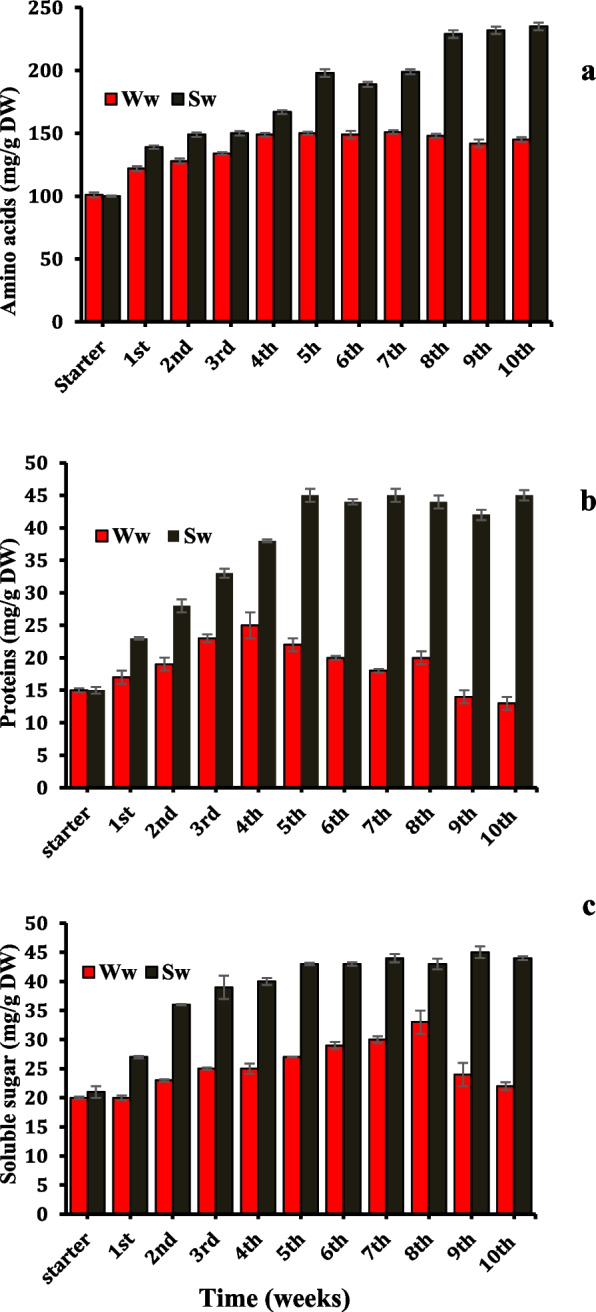
*Datura innoxia* leaves content of primary metabolites; amino acids (**a**), proteins (**b**), and soluble sugars (**c**) as affected by duration of well and sewage water irrigation (weeks). The data are averages of 4 replicates ± SE

The data presented in Figs. (1a, b, c) denoted that SW irrigation influenced the primary metabolites of *Datura* plants. Amino acids, protein, and soluble sugars content were accumulated by the irrigation of SW and reached the maximum values in plant leaves after the 5th week of irrigation with percent increases amounting to 32, 105, and 59%, respectively, over the corresponding values recorded for WW irrigated plants and maintained the highest values up to the end of the experiment; thereby, improving final plant growth.

#### *Datura innoxia* Nitric Oxide (NO) and Nitrate Reductase (NR) Activity Under Sewage Water Irrigation

**Fig. 2:**
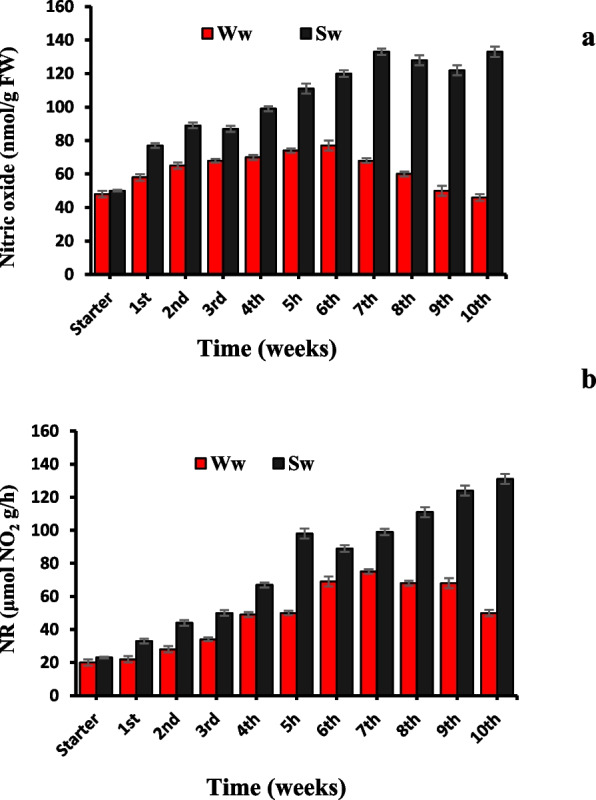
*Datura innoxia* leaves content of nitric oxide, NO (**a**) and nitrate reductase activity, NR (**b**) as influenced by duration of well and sewage water irrigation (weeks). The data are averages of 4 replicates ± SE

NO content of the leaves of SW-irrigated plants was elevated across the time of application with a maximum percent increase of 166% over the corresponding starter value (50 nmol/g FW) (Fig. 2a). Likewise, NR enzyme activity (Fig. 2b) was maximized by SW irrigation to be maximally recorded with percent increase of 470 over the corresponding starter value (23 μmol NO_2_ g/h) while their content kept low fluctuation and diminished at the end of the experiment under WW application with percent increase of only 54 and 120%, respectively.

#### *Datura innoxia* Secondary Metabolites: Anthocyanins, Flavonoids, Phenolics and Alkaloids and Activity of Their Mediated Enzyme, Phenylalanine ammonia lyase, Under Sewage Water Irrigation

SW irrigation noticeably increased secondary metabolites in terms of anthocyanins, flavonoids, phenolic compounds, and alkaloids recording final values with a percent increase of 81, 50, 19, and 37%, respectively, when compared to the corresponding values of WW irrigated plants (Table 4). This augmentation in secondary metabolites accumulation significantly (*P* < 0.0001) increased over time. Data depicted in Table 4 demonstrated that the interaction between water irrigations and time had a substantial impact (*P* < 0.0001) on secondary metabolites content.

The secondary metabolites-mediating enzyme, PAL, reached the maximal (122 μmol/mg protein/min) on the 10th week with a percent increase of 110% over the starter value (Fig. 4d), whereas the maxima under WW irrigation were on the 6th week recording value of only 81 μmol/mg protein/min which tended to decrease to reach a value of 70 μmol/mg protein/min.

#### *Datura innoxia* Stress Markers and Membrane Damage Criteria Under Sewage Water Irrigation

Some more indicative physio-biochemical parameters, such as hydrogen peroxide (H_2_O_2_, a stress marker) and membrane damage were evaluated as the levels of lipid peroxidation (malondialdehyde), as well as variations in enzymatic antioxidant capacities of *Datura innoxia* leaves, were monitored over a period of ten weeks for insight evaluation of the performance of *Datura* plants under different irrigations. Data in **Fig.** (**3****a-b**) revealed that irrigation of SW did not affect hydrogen peroxide (H_2_O_2._). Conversely, MDA concentration was reduced considerably submitted the lowest values (7.04 μmol/g FW) at the 6th and the 7th week.

**Fig. 3:**
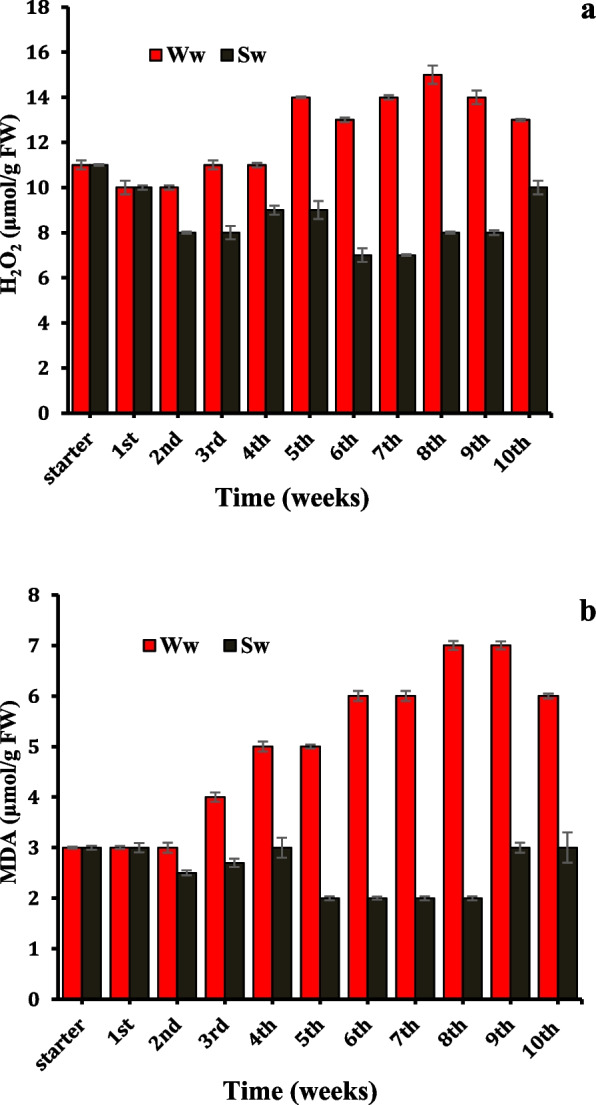
*Datura innoxia* leaves content of hydrogen peroxide, H_2_O_2_ (**a**) and malondialdehyde, MDA (**b**) as influenced by duration of well and sewage water irrigation (weeks). The data are averages of 4 replicates ± SE

#### *Datura innoxia* Specific Antioxidant Enzymes Activity Under Sewage Water Irrigation

**Fig. 4:**
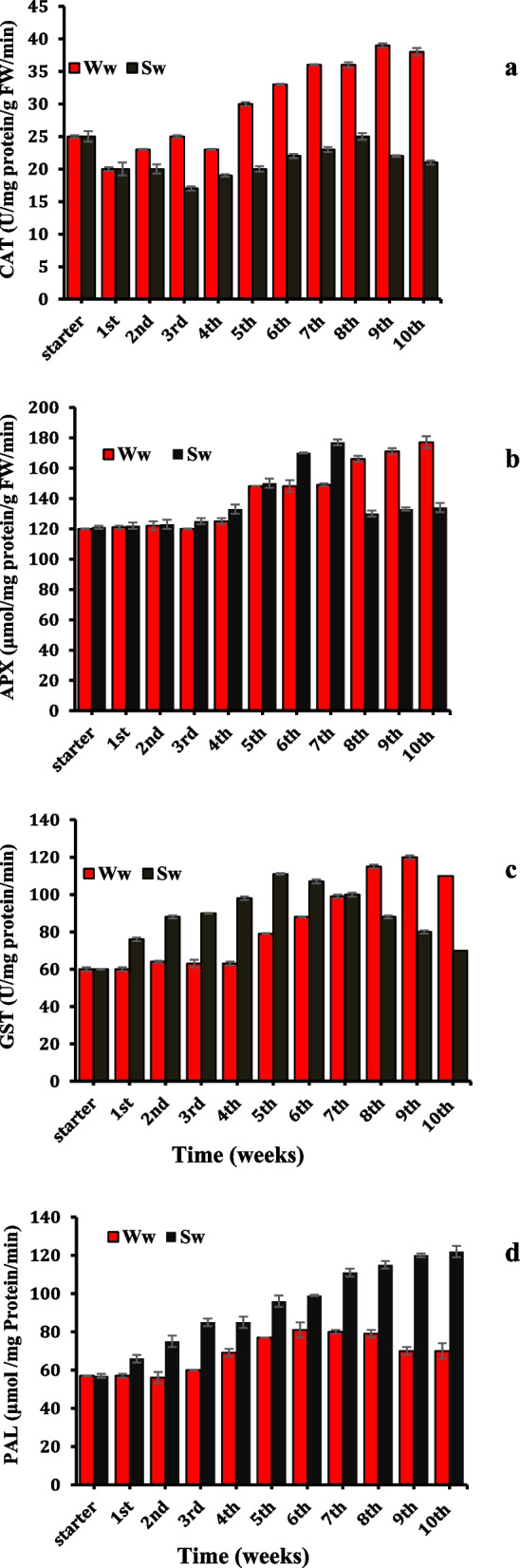
Alternations in the capacities of enzymatic antioxidant of *Datura innoxia* leaves; CAT, catalase (**a**), APX, ascorbate peroxidase (**b**) and GST, glutathione-S-transferase (**c**) and the activities of PAL, phenylalanine ammonia lyase (**d**) as influenced by duration of well and sewage water irrigation (weeks). The data are averages of 4 replicates ± SE

The enhanced related scavenger enzymes viz. After four weeks of irrigation, CAT, APX, and GST were concomitant with inducing cellular reactive oxygen species under WW application (Fig. 4a, b, c). The contrast was observed for SW irrigated plants where constant H_2_O_2_ and MDA content were kept around the control values as well as not affected by the screened activities of CAT and APX. However, GST had a divergent trend. It was activated in the case of SW application (Fig. 4c) as a defensive mechanism alarming the cell.

#### *Datura innoxia* Leaf Nutritional Profile Under Sewage Water Irrigation

Noticeable accumulation of macro- (Nitrate, Phosphate, K, Mg, and Ca) and micronutrients (Na, Fe, Zn, and Mn) in *Datura* leaves manifested in SW irrigated plants compared to WW irrigated ones (Table 5), with a percent increase, accounted by 43, 118, 48, 26, and 34% for nitrate, phosphate, K, Mg, and Ca, respectively, and 100, 71, 144 and 27% for Na, Fe, Zn, and Mn, respectively, when compared to WW irrigated plants.

**Table 5:** Leaf nutritional composition at the end of the experiment as affected by well and sewage water irrigations

Parameter (mg/g dw)	Well water	Sewage water	*P* Value
NO_3_	23 ± 1.29	33 ± 1.38	0.0016
PO_4_	0.87 ± 0.04	1.9 ± 0.06	< 0.0001
Na	11 ± 0.91	22 ± 1.11	0.0002
K	23 ± 1.78	34 ± 1.11	0.0017
Mg	4.6 ± 0.23	5.81 ± 0.41	0.0382
Ca	3.5 ± 0.21	4.7 ± 0.29	0.0122
Fe	7 ± 1.11	12 ± 1.11	0.0189
Zn	0.9 ± 0.03	2.2 ± 0.44	0.0229
Cu	0.2 ± 0.01	0.4 ± 0.05	0.0024
Mn	78 ± 0.85	99 ± 3.57	0.0012
Cd	nd	nd	–
Pb	nd	nd	–

*P*-values: *P* < 0.05 indicates significance, *P* < 0.01 indicates highly significance

## Discussion

Water resource scarcity is a vital problem in several localities in Egypt; thus, recycling and the reuse of alternative resources is a potential solution for getting rid of that massively produced SW. Furthermore, irrigating of certain plants is a good substitution for highly cost recycling treatment; however, the over-use on land for irrigation practices imposes considerable hazards causing chemical pollution problems. However, the plant growth and physio-biochemical parameters evaluated in the current study for along ten weeks showed an enrichment response rather than a toxic one, which can be due to the SW proprieties normally varying according to its locality and the source from which it is produced [17]. Additionally, pH value is highly effective in the mobility and bioavailability of various minerals [18]. As shown in Table (3) EC concentration in sewage water was higher (0.80 ds/m) than that of well water (0.51 ds/m), this is usually due to Cl and Na ion accumulation in SW but still within the recommended range. It was recommended that water with values of EC greater than 300 ds/m is unsafe for irrigation [17]. The SW herein has a considerable amount of carbon, potassium, and magnesium, essential nutrients for improving plant growth, soil fertility, as well as levels of productivity. These results were confirmed by the data reported by Chopra and Pathak [19]. However, in this research, HMs, as well as micronutrient concentrations in the SW, were considerably low, and they were within the limits of standard concentrations and recommended by [20], thus expected to be not harmful and to fulfill SW reuse standards in irrigation [19]. Contrarily, some previous studies reported high contents of some elements and HMs in SW [18], demonstrating the accumulation of these metals in plants as well as soil with continual SW usage in irrigation. Soil’s physicochemical properties indicated the poor quality of the used soil from Wadi El- Assiuty, aligning with the results of Attia et al. [11], who revealed that the presence of CaCO_3_ (counted as 32% of total salt in the soil) caused soil alkalinity in many regions of Wadi El- Assiuty and the OM, total nitrogen and total phosphorus content ranged between 0.019–0.73%, 30–370 mg/kg and 2.2–10.7 mg/kg, respectively. This finding may be because of ammonium nitrification as well as organic compound oxidation [17]. The current study indicated that after sewage irrigation, soil alkalinity decreased. These results are in accordance with Balkhair and Ashraf [21], who reported that SW treatment decreased pH. The increment in soil EC herein by the application with SW rather than WW could be ascribed to the original high level of total dissolved salts (TDS) of the sewage water as similarly reported by Mohammad and Mazahreh [22]. The current results revealed that irrigation with sewage water increased soil organic matter which is ascribed directly to the contents of the organic compounds and nutrients in the sewage water applied. Also, these results also agreed with those reported by da Silva et al. [23] who stated that sewage water irrigation considerably increased the soil OM, EC, N, and OC and diminished the soil pH. Several researchers attributed the accumulation of OM, N, and P in the soil with SW application to the original contents of these nutrients in the SW applied [24]. Furthermore, SW can result in K, P, and N in amounts equal to 4, 10, and 8 times the forage fertilizer requirements [25]. Similar results by Demir and Sahin [26] indicated that SW treatments increased soil fertility by elevating the micro-and macronutrients as achieved in the present work (Table 3). Contrarily, Mohammad and Mazahreh [22] and Mojid et al. [27] revealed an elevation in soil Fe and Mn with SW irrigation and no response regarding to Zn and Cu in the soil.. Regarding the most environmental hazards elements viz. Pb and Cd [28], the current study revealed that Pb and Cd were not affected by SW irrigation. Similar results obtained by Mohammad and Mazahreh [22], who mentioned that irrigation with SW had no substantial impact on the soil Pb and Cd concentrations.

Ten-week period was the limit of our study as the further application of WW completely stressed the plants grown in poor desert soil in terms of high values of ROS, alarming antioxidant capacity and decreasing primary metabolites manufacturing. Interestingly, SW irrigation exhibited substantial plant liveliness than WW irrigated plants, in addition to safely promoting characteristics of plants evaluated and continued for the 10 weeks. In this respect, the most substantial dry matter production and chlorophyll accumulation were recorded by SW application. This growth enhancement impact is compatible with Urbano et al. [29]. Some mineral ions in the irrigated SW, e.g., Cu and Mn, stimulate the two photosystems. Cu^2+^ enhances the rate of total electron transport from water to NADP. Mn^2+^ is essential for PSII (O_2_ evolving system). In addition, there is also a direct interaction between ferredoxin and copper in the reducing site of PSI [30]. Consequently, the stimulatory effect of SW on total chlorophyll could be ascribed to the SW impact, resulting in enhancing the rate of chlorophyll a and b biosynthesis. The elevated total chlorophyll content is parallel with the enhancing *Datura* plant biomass. Teixeira et al. [31] indicated a close correlation between biomass acquisition and chlorophyll content. These results are compatible with El-Okkiah [32], who found that the application of SW considerably increased protein and total carbohydrates content in Faba bean compared with WW irrigated plants. The manifested accumulation of nitrogen compounds in terms of amino acids and proteins in this study can be attributable to soluble, organic, or inorganic substances in the SW, that may collectively trigger growth [30]. Soluble sugar accumulation may indicate that SW-irrigated *Datura* plant leaves had the highest concentration of photo-assimilates, accelerating the transition from the vegetative to the flowering stage; consequently, flowering jointed with the triggering of photo-assimilate production in the green area, which was the primary source until reaching the reproductive. Chen and Chu [33] demonstrated that the content of nutrient components in SW had a decisive influence on *Ottelia acuminata* flowering, and the total number of flowers increased as nitrogen content in SW increased. The early flowering was supported by highly upregulated nitrogenous metabolism of the leaves, where proteins and amino acids accumulation as a result of SW irrigation suggest that the leaves are well-constructed and have an elevated metabolic efficiency [34]. Moreover, this nitrogen and carbon metabolite stimulation may provide the organic components necessary for forming new branches, where the most substantial carbohydrates, protein, and amino acids are linked to a greater number of branches per plant [34]. In the present study, sewage irrigated plants successfully submitted a nourished formation of lateral branches accounted by two folds that of WW irrigated plants. Song and Lee [35] reported that SW application caused shoot increase determined by new branch formation. In addition, this stimulation of carbon and nitrogen metabolites might accomplish the supplies of organic components required for forming new branches where the highest increase of proteins, carbohydrates, and amino acids corresponded to more branches per plant [34]. In the present study, sewage irrigated plants successfully submitted a nourished formation of lateral branches accounted by two folds that of WW irrigated plants.

Hence, the exacerbation of proline under WW irrigation across time was combined with the decrease in soluble protein generation. This apparent proline production was not always advantageous; rather, it may have been a negative consequence of extended WW irrigation of nutrient-poor soil. Göring and Thien [36] indicated that the proline content of plants increased at mineral nutrient deficiency, and in case of limited soil nutrition with prolonged WW and poor irrigation, plants may face nutrient deficiency-induced stress. Thus, this accumulation in proline content was considered an indicator of stress damage and/or stress resistance [36]. It might also act as a storage for organic nitrogen, which, upon stress reduction, could be converted into a variety of nitrogenous molecules [37]. The stabilization of proline content, combined with exacerbation in soluble protein content under SW application, may reflect optimum conditions for effective cellular metabolism and performance with SW irrigation.

Numerous studies indicated that NR substantially contributes to plants’ NO biosynthesis [38]. Therefore, in the current study, the activation of NR via SW irrigation was concomitant with the elevation of NO, which shoulders an essential role in plant immune signaling besides enhancing whole plant development [39]. Conclusively, SW irrigation might contribute to the activation of NR, thus influencing nitrate assimilation by supporting NR and its substrate (NO) uptake. Free amino acids, as well as proteins, were augmented due to the enrichment effect of SW irrigation upon catalyzing nitrogen assimilation enzyme NR [38].

Another healthy effect stimulated in SW irrigated *Datura* was the enhancement of secondary metabolisms and augmented production of highly valuable secondary metabolites that imparted the valuable medicinal properties and quality of *Datura* plant, viz. anthocyanins, flavonoids, phenolic compounds, and alkaloids. All of them meaningfully increased across the duration of cultivation owing to SW. It was suggested that SW irrigation improved de novo nitrogenous components synthesis, thus increasing the production of secondary metabolites compared to control [40].

Regarding *Datura innoxia* since it is a factory of interest gained alkaloids, it is noteworthy that the increase of alkaloids due to SW irrigation was concomitant with the low proline level along the study duration. This finding could be since ornithine, the precursor of tropane alkaloid, and the proline have the same precursor, namely: glutamic acid [41] that healthy directed to the pathway of ornithine production rather than accumulation of proline, the stress damage indicator. Ornithine was further profitably transported to torpane alkaloid, evident with the high content of the total alkaloids in *Datura* plants irrigated with SW [41]. Furthermore, the augmentation in the production of secondary metabolites was witnessed by enhancing PAL activities that reached the maximal on the 10th week with a percent increase of 110% over the starter value (Fig. 4d), indicating that SW irrigation effectively upregulated the production of secondary metabolites, along with elevated PAL activity. This result is mainly because PAL is an enzyme that synthesizes a precursor for the formation of different secondary metabolites and is a vital regulator between secondary as well as primary metabolism [37]. In contrast, the increased amino acid content may have improved the availability of phenylalanine (Phe) as an elite substrate for PAL, making more Phe available for the formation of secondary metabolites [37]. Contrarily, a low inducible PAL activity rate jointed with diminishing amino acid accumulation was pronounced under WW application, particularly in the last week of the experiment indicating down-regulation of the secondary metabolites production.

Whereas plants with WW irrigation exhibited elevated levels of H_2_O_2_ that were concomitant with decreasing membrane integrity and stability evidenced by high MDA concentration, which may be another main reason for suppressing the growth rate of *Datura* plants under WW irrigation compared to SW irrigated plants. As stress indicators, the enhanced levels of these toxic molecules (H_2_O_2_ and MDA) indicated that plants under prolonged WW irrigation might encounter nutrient deficiency after the fourth week and thus undergo nutrient deficiency-induced stress. Tewari et al. [42] reported that plants could undergo Zn deficiency-induced oxidative stress when poorly irrigated. As a response to this stress, plants may modify nutrition and metabolites in order to establish defense mechanisms on account of growth. Furthermore, the overproduction of anthocyanins, flavonoids, phenolic compounds, and alkaloids may partially explain the diminished lipid peroxidation in plants supplied with nutritive SW. Consequently, increased membrane integrity than the WW irrigated plants experiencing nutrient deficiency-induced stress. These valuable compounds display various functions jointed to antioxidant characteristics as well as the capacity to trap free oxygen radicals, thus stabilizing membranes by diminishing their fluidity, ultimately limiting free radical diffusion and reducing membrane lipid peroxidation [37].

The constant H_2_O_2_ and MDA content maintained near the control values reflects the high optimal condition and maintained membrane function for effective cellular performance as well as metabolism that can be attributed to the stabilized cell redox status [43], leading to a healthier growth of SW irrigated *Datura*. Moreover, activating GST as a defensive mechanism alarming the cell may be due to some xenobiotic agrochemical loaded in the delivered SW, which may trigger wide disciplines of antioxidants and metabolic pathways that cumulatively improve leaf physiological status in *Datura* plants. Hence, SW irrigation positively influenced the growth and physiological parameter as well as valuable secondary metabolites production of *Datura*.

However, SW irrigation manifested a noticeable accumulation of macro- (Nitrate, Phosphate) and micronutrients (Na, K, Mg, Ca, Fe, Zn, Mn) in *Datura* leaves plants compared to WW irrigated ones. The contents were still within the critical limits recorded for the metals’ phytotoxicity [24]. Nitrogen (N) and phosphorous (P) are the main elements of plant nutrition [44] and are fundamental to plant development, growth, crop yield, and adaptation [45]. In this study, N and P levels increased in plant leaves (in the form of nitrate and phosphate) after SW application. Amâncio and Stulen [46] stated that nitrogen content is one of the vital factors influencing crop growth and determining the quantity and quality of crop yields. Furthermore, Mohammad and Ayadi [47] and Hernández-Pérez et al. [48] stated that SW increased nutrient uptake in the shoot system, thereby not only improved soil properties by enriching with essential nutrients but amplified the quality of *D. innoxia* plants [49] as well. Consistent with our results, Bedbabis et al. [50] suggested that SW application significantly increases K, P, N, and HMs (Mn and Zn) concentrations in the olive leaves, thereby upgrading the olive property. It should be noted that SW lessens the toxicity of some elements in the soil, as suggested by Demir and Sahin [26]. Therefore, the growth of *Datura* plant in the presence of SW indicated suspected resistance against HMs accumulated in the soil.

## Conclusion

The ten-week monitoring period was the timeframe/limit of our study as the further application of WW completely stressed the plants grown in poor desert soil in terms of high values of ROS and alarming antioxidant molecules and the dramatically diminished primary metabolites’ content. Optimistically, according to the revealed results regarding healthy growth and improved medicinal property of *Datura innoxia*, jointed with a well-furnished metabolic profile and positive antioxidative changes in response to SW irrigation, it can be deduced that SW could be safely reused for *Datura* cultivation while at the same time to provide poor soil with adequate amendments. Hence, the soil was fertilized by the nutrient favorable to plant performance and development rather than be diseased. Nevertheless, the existence of high concentrations of HMs and traces of some toxic compounds lead to the instance for continual monitoring of the redox status of the receiver plant and the feedback on its growth. In that case, it will have priority for irrigation purposes. Thus, experiencing the crises of water scarcity and costive fertilizers, sewage might be effectively generalized for irrigation multipurpose plants.

## Materials and Methods

### Water and soil samples collection

The experiment was conducted over ten weeks, from March to mid-May 2019. Two water samples were collected, one from Arab Elmadabegh, Assiut, Egypt (27°12 N and 31°09 E), where the most extensive sewage line in Assiut governorate [51] (source of SW), another one was collected from tape water (source of WW) at the botanical farm of Botany and Microbiology Department, Faculty of Science (42 “and 28° 59’ 23 “E and latitude 25° 45` 06 “and 25° 53’34 “N). As described in the following section, the two water samples were analyzed for their physicochemical characteristics (Table 1) and were directly kept in the dark bottles under cooling (4 °C) for further usage in irrigation. Soil samples were collected from the surface soil at 0–25 cm soil depth from Wadi Al-Assiuty (31°18′ and 31°48′ E and 27°10′ and 27°45′ N), a part of the eastern desert east of Assiut city that has been recorded as a poor desert soil area in Assiut, Egypt [11]. The samples were air dried, ground to pass through 2 mm sieve, and stored in plastic bottles before usage. Soil samples were analyzed for the physicochemical characteristics two times, one prior to the start of the lab experiment and another at the end of the experiment, and plant harvesting results were represented in Tables 2 and 3, respectively.

### Physical and chemical analysis of the samples

For both water and soil, soluble Ca and Mg concentrations were measured using the EDTA titration method, and Na and K were estimated using a flame photometer. OC for soil was evaluated by adopting the method of Jackson [52]. Soil total nitrogen was determined following the procedure of Singh et al. [8]. Soil phosphorus was determined using Olsen extraction (0.5 M NaHCO_3_) [53]. Cation exchange capacity (CEC) was as described by Jackson [52]. Free calcium carbonate (CaCO_3_) was estimated by calcimeter method [54]. Available micronutrients and HMs were estimated as per the procedure described by Singh et al. [8]. The pH of water and soil (1:1 suspension) was estimated according to McNeal [55]. Electrical conductivity was measured using a conductivity meter (Orion, EA 940 USA). Soil texture was analyzed as the method described by Piper [54]. Water and soil samples were examined for different physical and chemical characteristics as per the standard procedure depicted in Tables (1, 2, and 3).

### Growth Condition and Treatments

The experiment was performed during spring 2019. Plants were gathered from the botanical garden of the Faculty of Agriculture, cut into 160 uniform/same-sized plantlets of approx. 25 g. Plantlets were randomly divided into two equal groups; WW and SW irrigations. Afterward, plantlets were washed with fresh water and weighed before being transplanted into the pots. The pots (60 cm in diameter and 45 cm in depth, filled with 50 kg desert soil collected from Wadi Al-Assiuty) were organized in a completely random arrangement with four replicates for each group, and plantlets were transplanted at the rate of 20 plantlets/pot. Immediately, they were transported to a greenhouse at the Department of Botany and Microbiology, Faculty of Science, Assiut University, receiving natural light (transmitted through glass panels) under ambient sunlight, the temperature ranging between 27 and 38 and 12–15 °C at day and night, respectively; relative humidity (34–42%) and reference evaporation (4.65–5.48 mm).

At the beginning of the experiment, using watering can, the dry soil received the WW and SW in field capacity at the rate of 180 ml/kg, soil with polyethylene bags to avoid soil treatment leaching. During cultivation, the frequency of SW was maintained once a week as per the plant water requirement (compensating the lost water via evaporation and maintaining the moisture level at field capacity). Along with experiment duration, soil water content was sustained in field capacity by weight method via the addition of WW day by day if required.

The harvest of all treatments was scheduled at 7-day intervals and performed by picking up the whole plant, root and shoot, from the soil. At the end of each week and for ten weeks, the observations were recorded on 2 randomly selected plants per replication per treatment for all indices. For the chemical analysis, 2 randomly selected plants were handled by blending as one sample.

### Growth Parameters and Physio-Biochemical indices Analysis of *Datura innoxia* Plant

#### Growth Analysis

Fresh weight (FW) of harvested plants was determined immediately and then cleaned via thoroughly rinsed with distilled water to be oven dried at 60 °C to constant weight for two days to evaluate DW. The number of branches and flowers per plant was also calculated.

#### Chlorophyll Content

Chlorophyll was extracted from 0.5 g of fresh leaves suspended in 5 ml of 95% ethyl alcohol at 60–70°C in a water bath. Absorbance readings were taken with a spectrophotometer (Unico UV-2100 spectrophotometer). Chlorophyll was estimated as mg/g FW at 663 and 644 nm using equations of Lichtenthaler [56].

#### Nitric Oxide Content and Nitrate Reductase Activity

NO content was quantified according to Ding et al. [57] and Hu et al. [58] and expressed as nmoles/g FW. Leaves were incubated in a buffer of acetate (pH = 3.6), and the leaves tissue was then separated by centrifugation and re-extracted by charcoal, then centrifuged again, the supernatant was mixed with Greiss reagent and read at 540 nm.

NR activity expressed as micromoles of NO_2_ g/hr. was estimated by adopting the described method of Downs et al. [59], in which leaves were soaked in potassium phosphate buffer (pH 7.5) and KNO_3_. The resultant Nitrite was detected by adding naphthyl-ethylenediamine dihydrochloride and sulfanilamide. Absorbance readings at 540 nm were taken with a spectrophotometer (Unico UV-2100 spectrophotometer).

#### Determination of Primary Metabolites

First, 1 ml of Stannus Chloride reagent was combined with 0.5 ml of the water extract, and then the tubes were heated in a water bath for 20 minutes and then cooled. The plant water extract was made by steeping 0.5 g of dry leaves in 10 ml of distilled water for 1 hour at 95 °C. The extinction of violet color was measured at 570 nm using the aforementioned reagents and distilled water instead of the extract of the plant sample [60]. Soluble protein was assayed according to Lowry et al. [61]. In the previous water extract of free amino acid, 0.1 ml of plant water extract was added to 5 ml of the alkaline reagent solution. Afterward, 0.5 ml of diluted Folin-Ciocalteu’s reagent (1: 2 v/v) was added. After 20 min, the extinction against the appropriate blank was measured at 750 nm utilizing a spectrophotometer. The water-soluble sugars were estimated by the method of anthrone–sulfuric acid according to the method of Fales [62] and Schlegel [63]. In addition, 30 mg of dry leaves were taken and extracted in 3 ml distilled H_2_O, which was blended with 4.5 ml anthrone reagent and boiled in a water bath for 5 min before cooling down on an ice bath. The absorbance of the developed blue-green color was determined at 620 nm using a Unico UV-2100 spectrophotometer. Proline was determined in dry leaves. Leaves tissue was ground in 6 ml sulfosalicylic acid (3%) before the centrifugation of the mixture. Then, the outcome supernatant was mixed with 2 ml of glacial acetic acid as well as 2 ml of ninhydrin. The reaction mixture was extracted with 4 ml toluene to quantify at 520 nm [64].

#### Determination of Secondary Metabolites

Determination of anthocyanin pigments was done according to the method described by Dawood and Abeed [65] on acidified methanol (1% HCl v/v) extract of fresh leaves that were hydrolyzed at 80 °C for 30 minutes to the absorbance obtained corresponding to anthocyanidins was spectrophotometrically detected at 520 nm. Determination of phenolic content was according to Kofalvi and Nassuth [66] using the Folin-Ciocalteu’s phenol reagent. Subsequently, 100 μl of the methanol extract was diluted to 1 ml with distilled water and mixed with 0.5 ml of Folin-Ciocalteu’s reagent (2 N) and 2.5 ml of Na_2_CO_3_ (20%). The absorbance of the developed color was measured at 725 nm with a Unico UV-2100 spectrophotometer. The methanolic extract of fresh leaves was utilized to analyze flavonoids by the method by Harborne and Williams [67]. Five ml distilled water and 3 ml AlCl_3_ (1:10) were added. After 5 min, 2 ml 1 M CH_3_- COOK was added, the total volume was made up to 10 ml, and absorbance was measured at 415 nm. A routine quantification method for analysis of the total alkaloidal content spectrophotometrically. The yellow-colored complex formed followed at 435 nm based on Dragendorff’s reagent (DR) described by Sreevidya and Mehrotra [68].

#### Oxidative Stress Indicators

The content of hydrogen peroxide (H_2_O_2_; μmol/g FW) was measured spectrophotometrically in the leaves. Fresh leaves were ground in cold acetone (5 ml). Afterward, 3 ml of the acetone extract was added to 1 ml of titanium dioxide (0.1%) in H_2_SO_4_ (20%) before centrifuging the mixture at 6000 rpm for 15 min. The yellow color developed was measured at 415 nm [69]. Malondialdehyde as a lipid peroxidation marker (MDA; μmol/ g FW) was quantified utilizing the protocol of Madhava Rao and Sresty [70]. Fresh leaves were homogenized in trichloroacetic acid (TCA) (0.1%) and then centrifuged at 10,000 rpm for 10 min. One ml of the supernatant was mixed with a TCA-TBA reagent. The mixture was heated for 20 min in a water bath at 90°C and then cooled rapidly on an ice bath. The resultant mixture was centrifuged for 15 min at 10,000 rpm, and the absorbance of the supernatant was spectrophotometrically monitored at 532 nm.

#### Enzyme Extraction and Quantification for Antioxidant Activities

Plant samples (four replicates from each treatment) were extracted via homogenizing leaf samples in 0.1 M phosphate buffer (pH 7.4) containing 10 mM β-mercaptoethanol, 1 mM EDTA, and 1% polyvinylpyrrolidone. The homogenates were centrifuged at 10,000 *g* for 25 min, and the supernatant was used for the assays. The activities of catalase (CAT; EC 1.11.1.6) and ascorbate peroxidase (APX; EC 1.11.1.11), glutathione peroxidase (GPX/EC.1.11.1.9), glutathione-S-transferase (GST; EC 2.5.1.18), and (PAL; EC 4.3.1.5) were assayed following the method of Abeed et al. [71], Flohé and Günzler [72], Habig et al. [73], and Sykłowska-Baranek et al. [74], respectively.

#### Determination of Leaf Element Composition

**Fig. 5:**
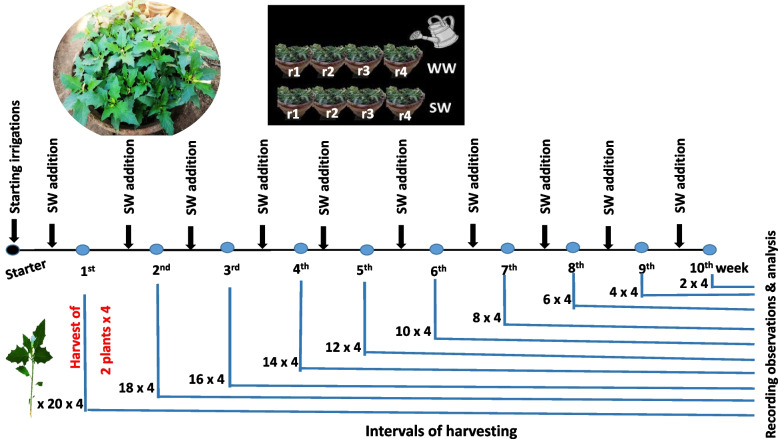
Summarization of the harvest intervals and data collected throughout the study from transplanting to finalizing. WW; well water, SW; sewage water

Potassium and sodium concentrations were measured utilizing the flame emission technique (Carl-Zeiss DR LANGE M7D flame photometer) according to Abeed and Dawood [75]. Nitrate content was determined following the protocol of Cataldo et al. [76]. Phosphorus content was spectrophotometrically measured by the methods of Fogg and Wilkinson [77]. The Ca, Mg, Fe, Zn, and Mn contents were determined with atomic absorption (Shimadzu- model AA-630-02) in acid-digestion extract (2:1 HNO_3_:HClO_4_ mixture), as described by Eissa and Abeed [78]. Summarization of the harvest intervals and data collected throughout the study from transplanting to finalizing is provided in Fig. 5.

### Statistical Analysis

The analysis of variance for completely randomized design (CRD) was carried out using Costat (CoHort software, Monterey, CA, USA) with two main treatments, WW and SW. The observations were recorded on plot mean basis analysis [79]. Means were compared by revised Least Significant Difference (R LSD) at a 5% level of significant [80].

## Data Availability

All the data is in the published article.

## Abbreviations

WW: Well water
SW: Sewage water
FW: Fresh weight
DW: Dry weight
SV: Starter value
BN: Branch number
FN: Flower number
TC: Total chlorophyll
Car: Carotenoids
An: Anthocyanin
FL: Flavonoids
Ph: Phenolics
Al: Alkaloids
Pro: Proline
NO: Nitric oxid
NR: Nitrate reductase
MDA: Malondialdehyde
ROS: Reactive oxygen species
H_2_O_2_: Hydrogen peroxide
CAT: Catalse
APX: Ascorbate peroxidase
GPX: Glutathione peroxide
GST: Glutathione-s-transferase
PAL: Phenylalanine ammonialyase
